# Differential modulation of post-antibiotic colonization resistance to *Clostridioides difficile* by two probiotic *Lactobacillus* strains

**DOI:** 10.1128/mbio.01468-25

**Published:** 2025-07-21

**Authors:** Matthew H. Foley, Arthur S. McMillan, Sarah O'Flaherty, Rajani Thanissery, Molly E. Vanhoy, Mary Gracen Fuller, Rodolphe Barrangou, Casey M. Theriot

**Affiliations:** 1 Department of Pathobiology and Population Health, College of Veterinary Medicine, North Carolina State University 70727, Raleigh, North Carolina, USA; 2 Department of Food, Bioprocessing and Nutrition Sciences, North Carolina State University 738941, Raleigh, North Carolina, USA; University of Maryland School of Medicine, Baltimore, Maryland, USA

**Keywords:** microbiome, *Lactobacillus*, probiotic, *Clostridioides difficile*, colonization resistance, *Muribaculaceae*

## Abstract

Probiotics are often consumed after antibiotic treatment to prevent antibiotic-associated diarrheal disease, most commonly caused by *Clostridioides difficile*. However, the impact of probiotic bacteria on the post-antibiotic gut microbiota is undetermined and often overlooked. Here, we examined the effect of a single dose of probiotic *Lactobacillus acidophilus* NCFM and *Lactobacillus gasseri* Lg-36 on colonization resistance against *C. difficile* in an antibiotic-treated mouse model. We found that *L. acidophilus* administration increased *C. difficile* infection and impaired the restoration of colonization resistance. In contrast, *L. gasseri* decreased *C. difficile* and promoted the return of colonization resistance, presumably through a putative bacteriocin inhibiting *C. difficile*. However, *L. gasseri* transiently colonizes the mouse gut, and its administration impacts colonization resistance after it is no longer detectable. We analyzed the gut microbiota of mice and found that members of the understudied *Muribaculaceae* family were enriched after *L. gasseri* administration and associated with colonization resistance. Using *Muribaculum intestinale* and *Duncaniella muris*, we determined that elevated growth of these species can restrict *C. difficile* growth *in vitro*, suggesting that these bacteria may play a role in establishing colonization resistance *in vivo*. These findings highlight the potential pitfalls of specific probiotic strains taken after antibiotic treatment and support the need for further investigations of their influence on the gut microbiota post-antibiotic. Additionally, this work supports the role of the *Muribaculaceae* as beneficial gut commensals that can contribute to colonization resistance against *C. difficile* and illustrates the need to decipher community interactions in complex microbial consortia.

**IMPORTANCE:**

Probiotic research has overwhelmingly generalized the safety of select strains perceived as beneficial, while most studies are based on individual strains to substantiate particular functional attributes. In contrast, *Clostridioides difficile* studies document how this complex pathogen interacts with diverse members of the gut microbiota to cause diarrheal disease. Despite their purported ability to inhibit pathogens and modulate the gut microbiota, probiotics have been used to treat *C. difficile* infections with little success. In this study, we examine how common probiotics can impact the recovery of the gut microbiota after antibiotics by measuring colonization resistance against *C. difficile* in a mouse model. We show that *Lactobacillus acidophilus* enhances *C. difficile* infection, while *Lactobacillus gasseri* promotes colonization resistance potentially through its expression of bacteriocins and an enrichment of *Muribaculaceae*. This work highlights the complexity of probiotic interactions with pathogens and the indigenous microbiota and further supports that the overlooked *Muribaculaceae* are capable of inhibiting *C. difficile*.

## INTRODUCTION

Probiotics are one of the most commonly consumed dietary supplements due to their purported health benefits and widespread availability (1, 2). Oral probiotic consumption as a result of personal preference and in response to a physician’s recommendation has dramatically increased in part due to the contention that live probiotic strains are regarded as safe and confer general health benefits, often centering around gut health, when administered in adequate amounts (3–5). The reported mechanisms by which these bacteria positively act on their host are numerous and include immunity and regulation of inflammation (6–10), beneficial metabolite production (11–17), digestion and metabolism of dietary compounds (18–20), promotion of gut mucosal barrier integrity (21–23), and modulation of the resident gut microbiota (12, 24, 25). While a body of research supports probiotic use for the prevention or treatment of many diseases (26), their beneficial effects are highly variable as a result of the differences between individual probiotics, their dosing, and the heterogeneity of individual gut microbiomes (27).

Probiotics offer an appealing strategy to target the gut microbiome for the treatment or prevention of disease given their interaction with both the host and the microbiota during its transit through the intestinal tract (28). While probiotics are assumed to strictly act in a beneficial manner, their capacity to modify gut microbiomes can have adverse effects if they introduce disruptive or unfavorable changes to the ecosystem (29). Moreover, the long-held notion that non-resident, autochthonous probiotics “balance” and modulate a resident microbiome to promote host health without exception is becoming increasingly challenged and seemingly naively over-simplistic (27, 30). This is exemplified in the case of antibiotic-associated diarrhea (AAD), a common adverse event following antibiotic treatment that significantly alters the gut microbiome resulting in diarrheal disease (31). Probiotics are often thought to counteract the negative consequences of antibiotic administration and prevent AAD by reconstituting and restoring the gut microbiome and increasing microbial diversity (32–37). However, this effect may not always be expected, and, in some cases, probiotics can impair or delay recovery of the gut microbiota after antibiotic treatment (38–41).

*Clostridioides difficile*, a gram-positive spore-forming enteric pathogen (42), is a significant cause of AAD, especially in healthcare settings (33). Antibiotic-induced perturbations to the gut microbiome result in a niche that supports the growth of *C. difficile* and the establishment of *C. difficile* infection (CDI) (43–45). Colonization resistance is the intrinsic ability of a healthy gut microbiome to inhibit *C. difficile* and is critical to prevent disease and resolve recurrent infections (46–48). While it is well known that colonization resistance is mediated by the gut microbiome (43–45), it is still not fully understood which microorganisms in the ecosystem determine this protection. For this reason, fecal microbiota transplants (FMTs; suspensions containing the entire fecal microbiome collected from a healthy donor) have served as an effective treatment for recurrent CDI, as they successfully reconstitute the gut microbiome and reinstate colonization resistance (46, 47, 49).

Given the complexity and safety concerns of FMTs (50, 51) and the challenges of live biotherapeutic products (LBPs) (49, 52, 53), probiotics still remain an attractive therapeutic option to prevent or treat CDI. Probiotics have the opportunity to directly inhibit *C. difficile* through bacterial antagonism or competition for nutrients (54, 55) and indirectly by modifying the microbiome in order to restore colonization resistance (12, 24, 25). Unsurprisingly, LBP and probiotic trials for the prevention of CDI have been met with mixed results (36, 52), though several meta-analyses have provided moderate evidence for their efficacy and safety in this patient population (56–59). Still, there is reason to believe that probiotic administration can have negative effects in this patient population and further complicate disease (29, 41). Given this evolving evidence, probiotics have not been recommended by the U.S. Food and Drug Administration as a companion therapy alongside the standard of care antibiotics, thereby underscoring the need to mechanistically understand the interplay between probiotics, the gut microbiome, and colonization resistance against CDI in order to inform our selection of current probiotic candidates and engineer the next generation of probiotic therapies.

Here, we investigate how two common probiotic strains (*Lactobacillus acidophilus* NCFM and *Lactobacillus gasseri* Lg-36) can affect the structure and function of the gut microbiota after antibiotic treatment by measuring the restoration of colonization resistance over 4 weeks in a mouse model of CDI. Overall, throughout the duration of our experiment, *L. acidophilus* increased *C. difficile* burden and delayed the return of colonization resistance, while *L. gasseri* decreased *C. difficile* burden and accelerated the return of colonization resistance. We examined how these strains can directly inhibit *C. difficile in vitro,* and we also explored the potential indirect impacts of *Lactobacillus* colonization on the murine gut microbiota as it recovers from antibiotic perturbation. Mice that received *L. gasseri* displayed a potentially coincidental bloom of *Muribaculaceae* species at week 3 that coincided with an accelerated return of colonization resistance. To see if the stochastic bloom of *Muribaculaceae* species could be responsible for the inhibition of *C. difficile*, we examined their competition for preferred carbohydrate nutrients. This study provides proof of concept that select probiotics provide specific beneficial functions to the host and that a deeper mechanistic understanding of probiotic strains is needed in different contexts, including through indirect modulation of the gut bacterial community.

## RESULTS

### Transient probiotic colonization of the murine gut impacts the return of colonization resistance after antibiotics

**Fig 1:**
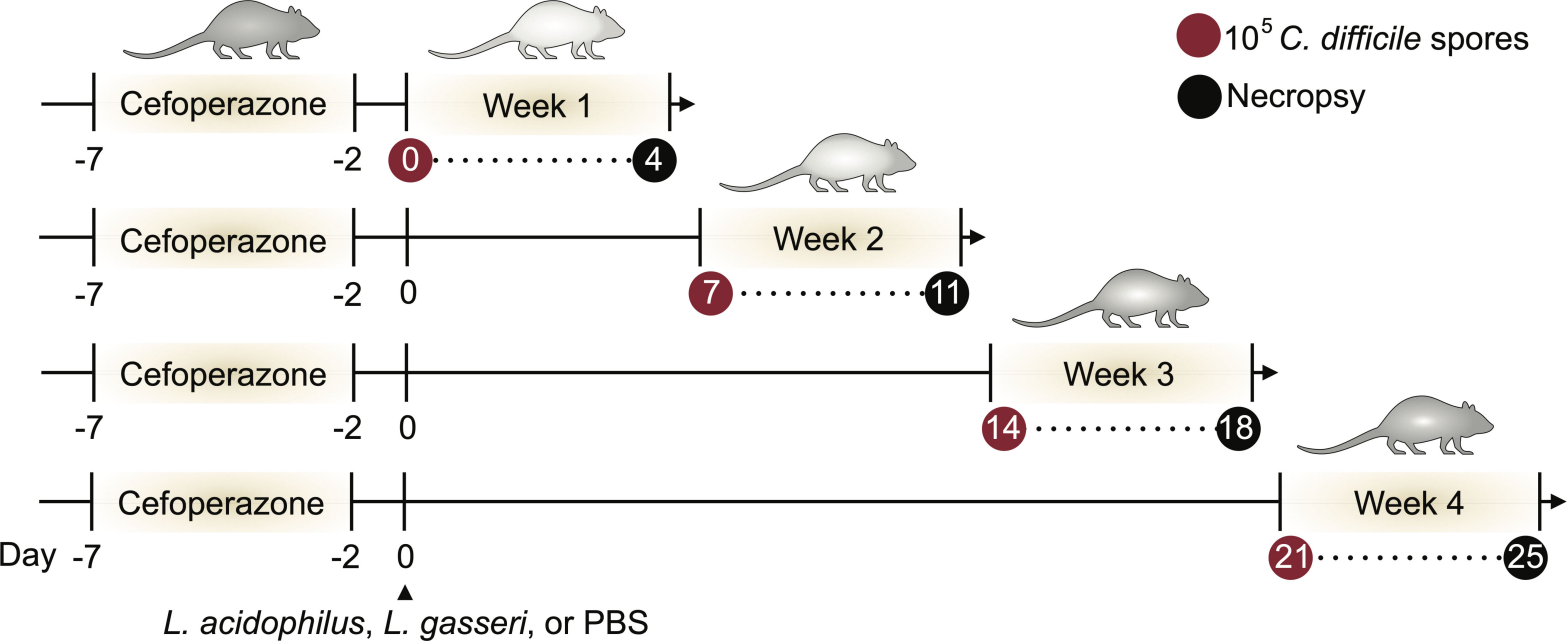
An antibiotic-treated mouse model that measures the return of colonization resistance after probiotic administration. Schematic of the mouse model monitoring colonization resistance after antibiotic treatment. All mice (*n* = 32) received cefoperazone in their drinking water for 5 days. Mice were orally gavaged at day 0 with *L. acidophilus* NCFM, *L. gasseri* Lg-36, or no probiotic following a washout period of 2 days. At day 0 and with each successive week, different subsets of mice were challenged with 10^5^ *C. difficile* R20291 spores, and clinical signs of CDI were measured over a 4-day period until mice were sacrificed, and intestinal contents were collected at necropsy.

**Fig 2:**
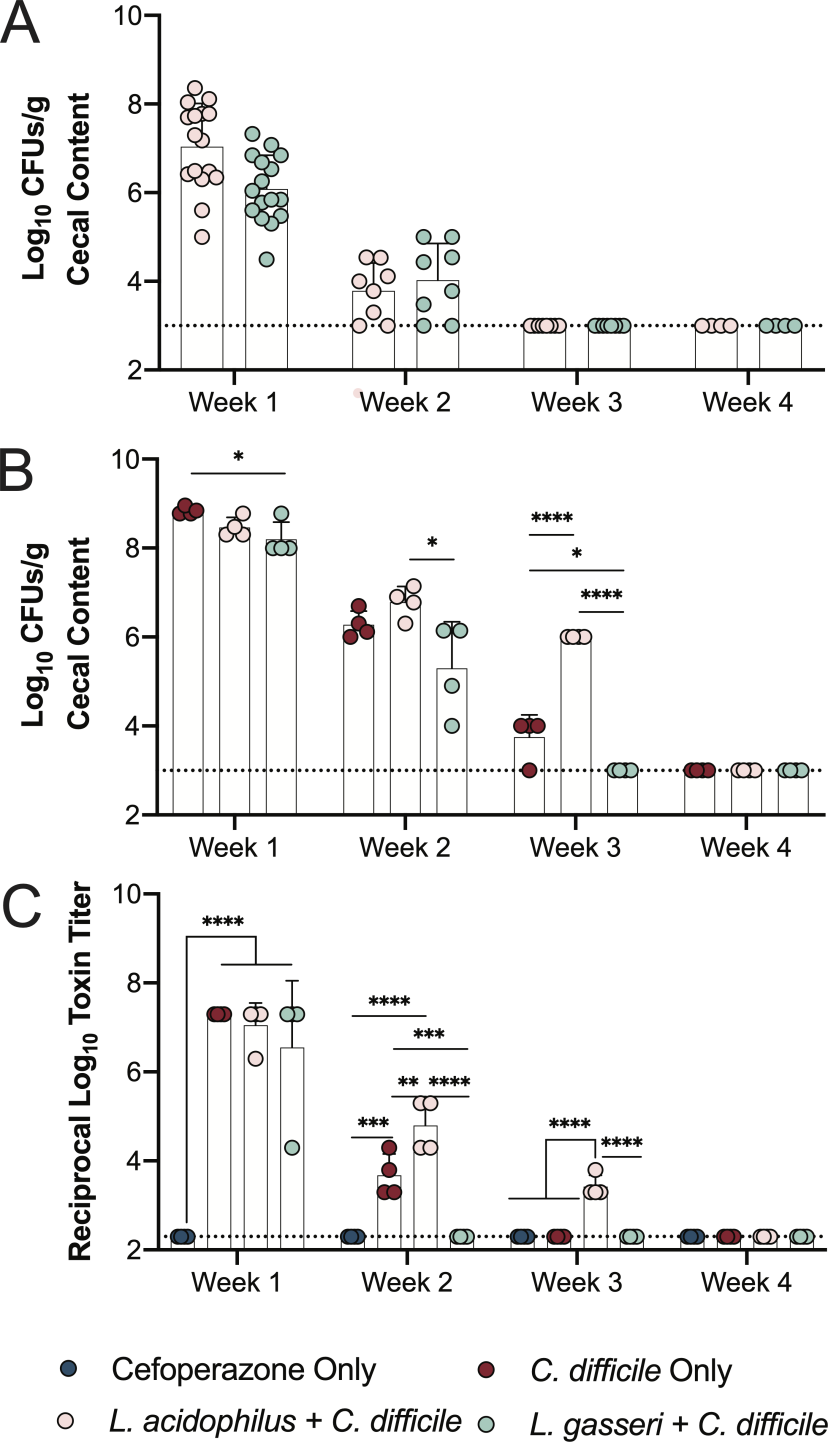
Probiotic *Lactobacillus* alters the return of colonization resistance against *C. difficile* after antibiotics. (**A**) Probiotic *Lactobacillus* CFUs (*L. acidophilus* and *L. gasseri* alone) collected from the feces of mice each week post-antibiotics. (**B**) *C. difficile* CFUs collected from the cecum of challenged mice each week post-antibiotics. (**C**) *C. difficile* toxin activity recovered from the cecum. All data presented as mean ± standard deviation from *n* = 4 replicates. One-way ANOVA with Tukey’s correction for multiple comparisons was used in B and C. *P  <  0.05, **P  <  0.01, ***P  <  0.001, and ****P  <  0.0001.

*L. acidophilus* NCFM and *L. gasseri* Lg-36 were selected as model probiotic strains to test due to the wide use and inclusion of these species in common probiotic formulations and their use to modulate CDI in pre-clinical and clinical trials (36, 60–63). A cefoperazone-treated mouse model of CDI was leveraged to measure how different probiotics affect the return of colonization resistance after antibiotics (Fig. 1) (64). Five days of treatment with the broad-spectrum cephalosporin antibiotic cefoperazone, followed by a 2-day washout period with no antibiotic, allows for reproducible changes in the murine gut microbiota and metabolome that supports *C. difficile* colonization and disease (44). Cefoperazone-treated C57BL/6J mice were challenged with either PBS (*n* = 32), 10^8^ CFUs of *L. acidophilus* (*n* = 16), or 10^8^ CFUs of *L. gasseri* (*n* = 16) once by oral gavage at day 0 (Fig. 1). Separate groups of mice were subsequently challenged with 10^5^ CFUs of *C. difficile* R20291 spores each week for 4 weeks. Each week, the *C. difficile* challenge was monitored for 4 days for clinical signs of disease (weight loss, *C. difficile* load, and toxin activity). All challenged mice were sacrificed, and intestinal contents and tissues were harvested at necropsy. *Lactobacillus* colonization was monitored over the course of the experiment by generating spontaneous rifampicin-resistant mutants (65). This permitted us to plate cecal contents and enumerate lactobacilli on selective LBS rifampicin plates (Fig. 2A). *L. acidophilus* and *L. gasseri* colonization reached initial levels of 10^7^ and 10^6^, respectively, and waned until they were no longer detectable by week 3.

*C. difficile* load was similarly monitored during necropsy by plating on selective TCCFA agar that supports the growth of *C. difficile* vegetative cells and spores (Fig. 2B). The *C. difficile* burden across treatment groups in week 1 was high, though *L. gasseri + C. difficile* mice contained a small but significant reduction in *C. difficile* CFUs. However, this reduction did not result in any difference in clinical signs of disease; all mice lost similar amounts of weight during the course of week 1 (Fig. S1), and histopathological change to the cecum did not reveal any differences due to *Lactobacillus* administration (Fig. S2).

Mice that were challenged with *C. difficile* in week 2 overall had a much lower burden of *C. difficile* relative to week 1 mice (Fig. 2B) and had no clinical signs of disease as a result of returning colonization resistance (Fig. S2). *L. gasseri + C. difficile* mice had a significantly lower burden of *C. difficile* compared to *L. acidophilus + C. difficile* mice, highlighting the diverging effects on the course of colonization resistance that the two probiotic species establish. This was further supported by week 3 where *L. gasseri + C. difficile* mice did not have any detectable *C. difficile* at necropsy despite being challenged with the pathogen (Fig. 2B), suggesting that colonization resistance was fully restored. This is especially striking given the observation that the *C. difficile*-only mice supported a low level of *C. difficile* at this time. In contrast, the *L. acidophilus + C. difficile* still supported 10^6^ CFUs/g of *C. difficile* colonization that was significantly greater than the mice that received *C. difficile* only but was still overall lower than the previous 2 weeks (Fig. 2B). By week 4, there were no detectable levels of *C. difficile* in mice following the challenge with the pathogen, indicating that colonization resistance was fully restored in all groups and that this experimental timeframe was sufficient to capture a range of phenotypes surrounding the return of colonization resistance.

All week 1 mice that were challenged with *C. difficile* with or without *Lactobacillus* administration showed acute signs of disease by weight loss and significant histopathological changes to the cecum (Fig. S1 and S2). In order to better define the kinetics of CDI during our experiment, we measured the total amount of *C. difficile* toxin in mice ceca by quantifying toxin activity using a Vero cell cytotoxicity assay (Fig. 2C) (66). Toxin activity from week 1 mice challenged with *C. difficile* only was high, reflective of the high *C. difficile* burden shared across these mice. Similarly, the week 2 and 3 toxin activities mirrored the respective *C. difficile* burdens present in mice at those timepoints. *L. acidophilus* administration resulted in increased toxin activity, while *L. gasseri* resulted in decreased activity relative to *C. difficile* only at weeks 2 and 3. While the overall toxin loads at these timepoints were not sufficient to create significant levels of histopathological changes between the groups (Fig. S2), they emphasize how different *Lactobacillus* strains can alter *C. difficile* burden and pathogenesis post-antibiotic administration.

### *L. gasseri* impacts colonization resistance through direct and indirect mechanisms

**Fig 3:**
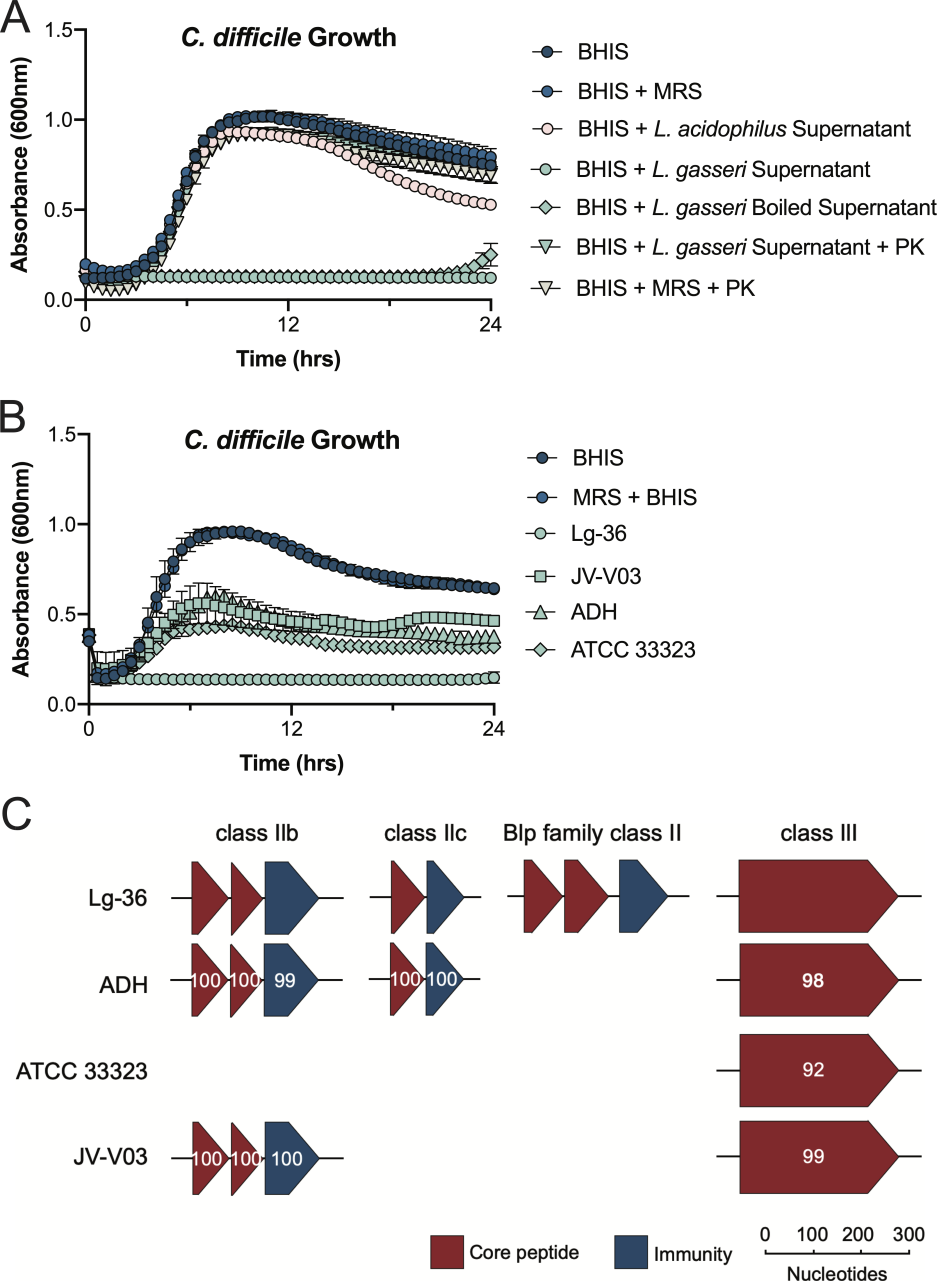
*L. gasseri* directly inhibits *C. difficile in vitro*. (**A**) Growth of *C. difficile* in BHIS supplemented with 10% MRS, *L. gasseri*/*L. acidophilus* supernatants, or enzymatic treatment. PK denotes proteinase K. (**B**) Growth of *C. difficile* with 10% *L*. *gasseri* supernatants from stationary-phase cultures. All data are plotted as averages ± standard deviation from *n* = 3 replicates. (**C**) Alignment of putative bacteriocin genes across *L. gasseri* strains predicted by BAGEL4 (67). Each protein was compared to Lg-36, and its percent amino acid identity is labeled within each gene.

Anti-pathogenic activity is a commonly sought-after probiotic attribute and often mediated by the production of antibacterial compounds that can contribute to colonization resistance by inhibiting intestinal pathogens (54). We assayed whether either *Lactobacillus* species could inhibit *C. difficile in vitro* by supplementing its growth in BHIS with neutralized spent MRS media (Fig. 3A). While *L. acidophilus* supernatants did not meaningfully impact *C. difficile*, *L. gasseri* supernatants completely inhibited its growth. We further investigated this effect by probing into the nature of the soluble inhibitory compound. Boiling *L. gasseri* supernatants did not significantly alter the inhibitory activity; however, proteinase K treatment completely abolished inhibition (Fig. 3A).

Based on the observations that this phenotype is mediated by a proteinaceous and heat-stable molecule, we predict that a soluble bacteriocin or antimicrobial peptide is responsible for this inhibition (68). To see how penetrant this phenotype is in other *L. gasseri* strains, we measured the inhibitory effects of *L. gasseri* strains, JV-V03, ADH, and ATCC 33323 and the rifampicin-sensitive parent strain, Lg-36 (Fig. 3B). Lg-36 completely inhibited *C. difficile* as expected, while the other strains displayed an intermediate level of inhibition, suggesting that this is something shared within the species. *L. gasseri* harbors several bacteriocins termed gassericins (69–72), which can be similarly heat-stable and proteinase-sensitive (72–74). To explore the presence and diversity of bacteriocins expressed by the *L. gasseri* strains included here, we mined their genomes for inhibitory peptides using BAGEL4 (67). Lg-36 encoded four distinct loci-harboring several class II (heat-stable) and one class III (heat-labile) putative bacteriocin genes (75), which were more than any of the strains analyzed (Fig. 3C). Lg-36 uniquely encoded for a Blp-like operon, a family of heat-stable peptides typically found in streptococci (76, 77). The exclusive collection of bacteriocins harbored by Lg-36 could work together to provide an explanation for its ability to effectively inhibit *C. difficile*.

While *L. gasseri* may be promoting colonization resistance at weeks 1 and 2 by directly inhibiting *C. difficile*, *L. gasseri* was no longer detected at week 3, making it unlikely that direct inhibition played a role at this timepoint. Therefore, we hypothesized that any colonization resistance resulting from *L. gasseri* administration by week 3 would likely be the result of its effects on the microbiota. We performed 16S rRNA amplicon sequencing on cecal DNA collected at necropsy (days 4, 11, 18, and 25), as well as fecal DNA collected on days that mice were challenged with *C. difficile* spores (days 0, 7, 14, and 21). We characterized the amplicon sequence variants (ASVs) present on the day of the *C. difficile* challenge and after the challenge.

**Fig 4:**
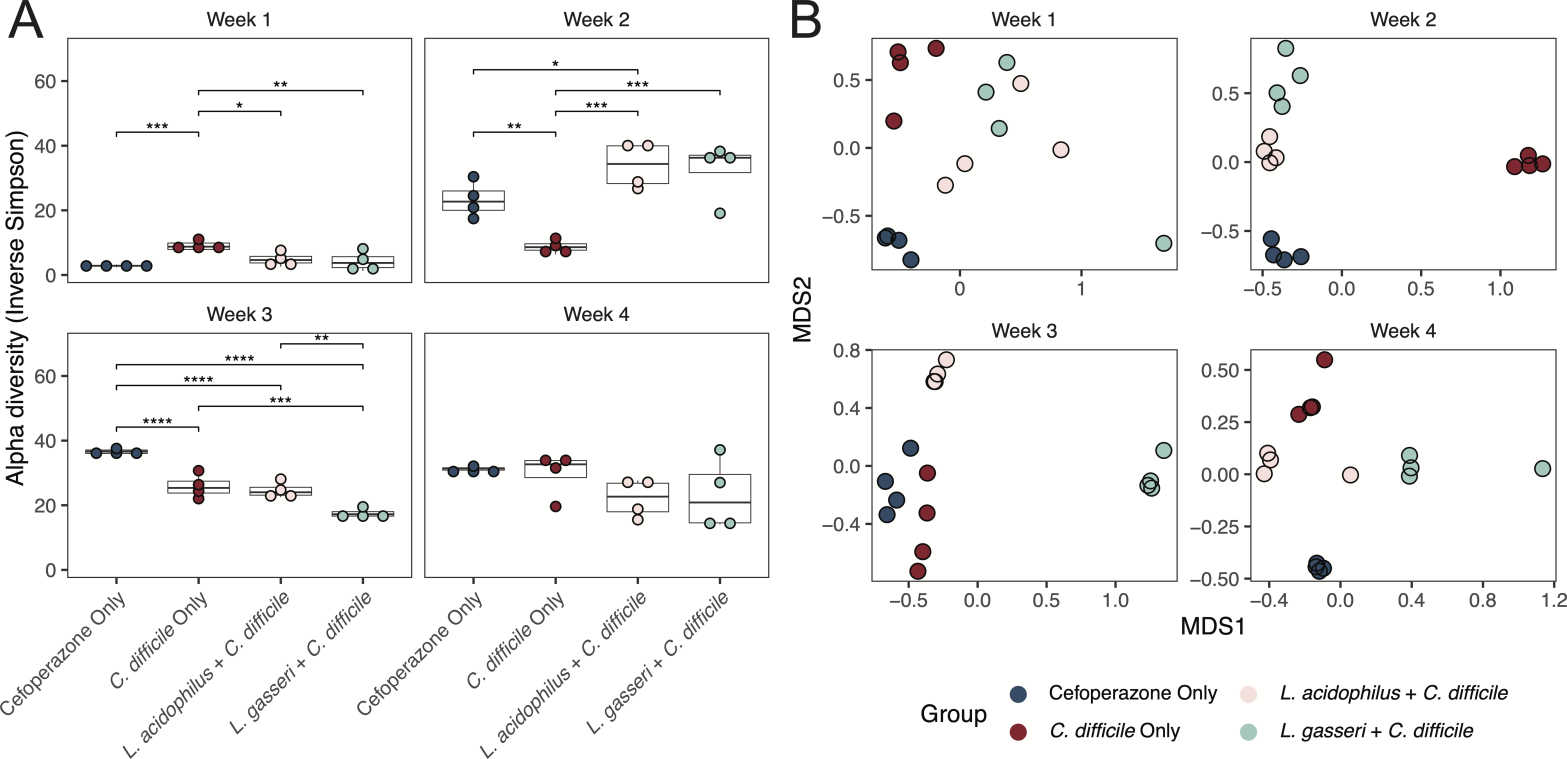
*Lactobacillus* administration alters microbial diversity after *C. difficile* challenge. (**A**) Inverse Simpson index of ASVs in the cecal microbiota presented in box and whisker plots for each week post-antibiotics. Kruskal-Wallis *P* values are listed in the top corner of each graph and used to determine statistical significance (*P  <  0.05, **P  <  0.01, ***P  <  0.001, ****P  <  0.0001) by Dunn test with FDR correction. (**B**) Bray-Curtis dissimilarity of cecal ASVs between samples plotted by NMDS. The stress of each NMDS is listed in the top corner of each graph.

Increased species richness measured as alpha-diversity in the gut microbiota is associated with a healthy microbial ecosystem and with colonization resistance against *C. difficile* (55). Alpha-diversity of the gut microbiota is known to decrease after antibiotics and increase as the microbial ecosystem recovers after antibiotic cessation (41, 78). Accordingly, we saw this trend in the fecal microbiota with each successive week (Fig. S3A). The fecal alpha-diversity of all groups increased at approximately the same rate, suggesting that *Lactobacillus* administration did not alter the fecal microbiota speed of recovery following antibiotics. However, when we measured the cecal alpha-diversity following *C. difficile* challenge, we found significant differences between the groups in weeks 2 and 3 but not in weeks 1 and 4 (Fig. 4A). While the mice that received *Lactobacillus* had the highest alpha-diversity at week 2, they subsequently had the lowest diversity in week 3. Notably, *L. gasseri + C. difficile* mice had the lowest alpha diversity in week 3 despite them displaying colonization resistance and were significantly different from the cefoperazone control. These data indicate that species richness may not always be predictive of colonization resistance. Rather, the compositional differences in the microbiota, including specific species that contribute to colonization resistance as well as the interactions of those bacteria with the larger microbial community, are important for predicting the colonization resistance status.

To examine the different groups’ microbiota each week, we determined and visualized the Bray-Curtis distances on non-metric multidimensional scaling (NMDS) plots. The fecal microbiota of the mice prior to the *C. difficile* challenge did uniquely cluster as a result of their *Lactobacillus* treatment (Fig. S3B), highlighting the differences in the microbiota’s reconstitution following cefoperazone treatment and reinforcing the notion that probiotics can alter the microbiota after antibiotic perturbations. Likewise, the cecal microbiota of mice after the *C. difficile* challenge varied across treatments and timepoints (Fig. 4B), with the week 3 *L*. *gasseri + C. difficile* mice notably distinguishing themselves from the other treatment groups. While these data support our observation that *Lactobacillus* administration can impact the microbiota’s composition, even after bacterial colonization has become extinct, and that this impact may drive alterations in colonization resistance against *C. difficile*, it is also possible that initial differences and stochastic shifts in the murine microbiota can account for some of the results we observed.

### *Muribaculaceae* contributes to colonization resistance against *C. difficile*

**Fig 5:**
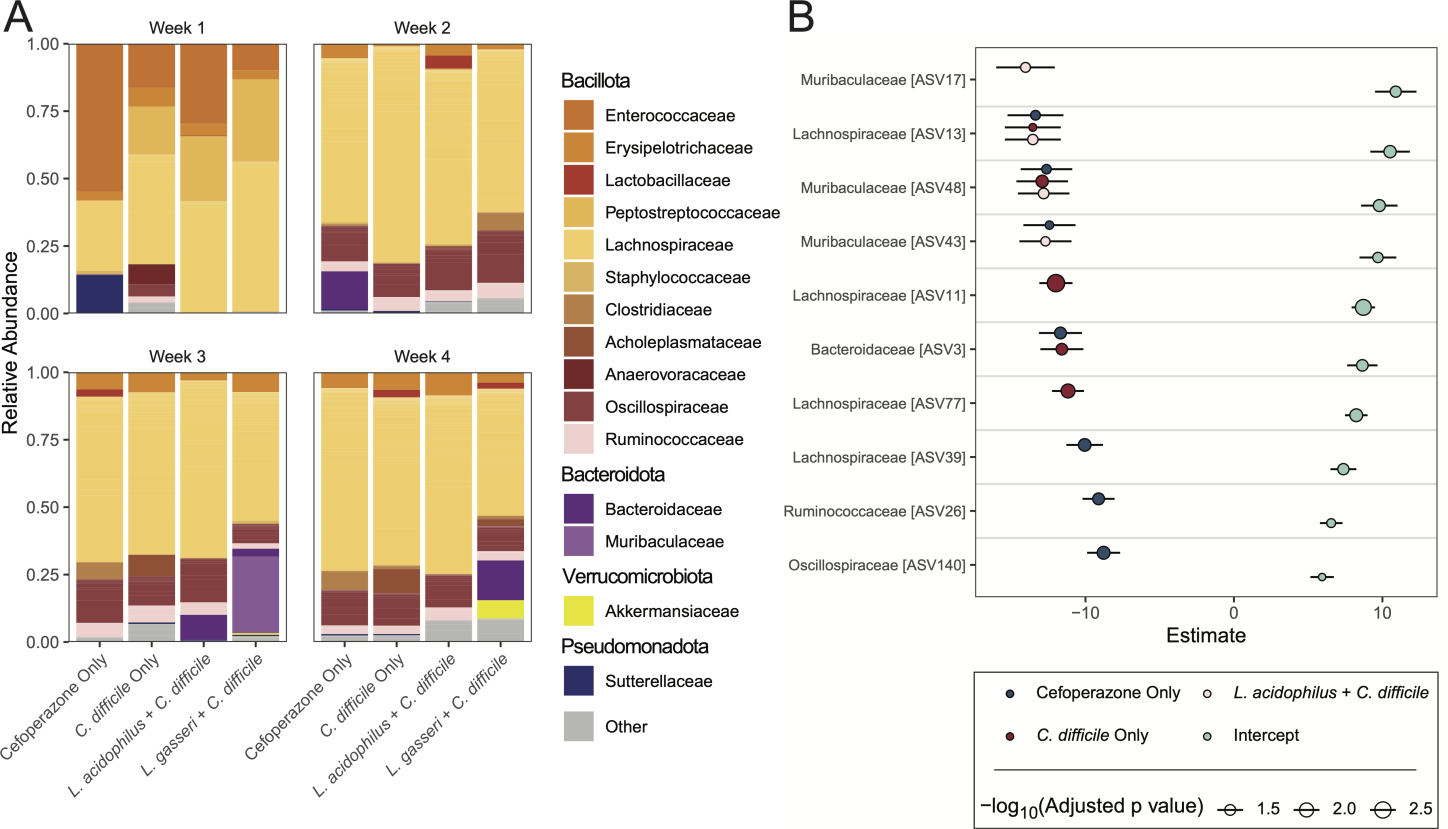
Probiotic administration results in unique taxonomic differences after antibiotic treatment. (**A**) Relative abundance of ASVs from the cecal microbiota grouped by bacterial families each week post-antibiotics. (**B**) Estimates obtained from generalized linear model with Holm correction of the center log ratio-transformed Monte Carlo Dirichlet instances obtained from ALDEx differential abundance analysis relative to ASVs in the *L. gasseri + C. difficile* group. The intercept serves as a reference for the *L. gasseri + C. difficile* group to show differences between this group and estimates from other groups. Only significant points (*P*-adjusted ≤ 0.05) are plotted.

Given that *Lactobacillus* colonization has been reported to alter the gut microbiota (41), we wanted to determine which microbial taxa were associated with the complete return of colonization resistance observed at week 3 in the *L. gasseri + C. difficile* mice. We plotted the average relative abundance of the bacterial families within the feces of mice prior to the *C. difficile* challenge and the cecum of mice after the *C. difficile* challenge each week (Fig. 5A; Fig. S4A). Gram-positive *Bacillota*, such as *Enterococcaceae* and *Peptostreptococcaceae* (of which *C. difficile* is a member), dominated the microbiota of mice at weeks 1 and 2. The week 3 microbiota were particularly distinguished from one another due to an increase of *Bacteroidaceae* in the *L. acidophilus* mice and *Muribaculaceae* in the *L. gasseri* mice relative to mice that received no *Lactobacillus* (Fig. 5A). It has been shown that members of the *Bacteroidaceae* can support the growth of *C. difficile*, perhaps explaining why *L. acidophilus* administration resulted in a higher burden of the pathogen (79). Both blooms did not result from the introduction of *C. difficile* since they were present in the feces prior to the challenge (Fig. S4A). However, we cannot rule out that these blooms occurred stochastically and independently of probiotic administration at this timepoint; the murine microbiota can vary due to cage-specific stochastic and founder effects despite controlling for mouse sex, age, experimental treatments, and other factors (80, 81).

Given the importance of understanding and identifying the beneficial bacteria that mediate colonization resistance (47), we performed a differential abundance analysis on the fecal and cecal microbiota using ALDEx2 to identify ASVs in the *L. gasseri*-administered mice that are uniquely abundant relative to the *C. difficile* only and *L. acidophilus + C. difficile* mouse cecal microbiota (Fig. 5B; Fig. S4B). ALDEx2-calculated ASV abundances were plotted and arranged by the largest difference between the *L. gasseri + C. difficile* intercept estimate and slope estimate of other groups, representing the largest change between groups. Many of the most differentially abundant ASVs were in the *Lachnospiraceae* family, which are known to contribute to colonization resistance against *C. difficile* (82). However, ASVs within the *Muribaculaceae* family (ASV17, ASV48, and ASV43) were among the most abundant after the *C. difficile* challenge, suggesting that these ASVs have the largest association with colonization resistance at week 3 (Fig. 5B). ASV17 was especially determined to have the largest intercept estimate in both the fecal and cecal microbiota of mice before and after the *C. difficile* challenge, respectively, indicating that this ASV strongly associates with the phenotype of colonization resistance seen in the week 3 *L*. *gasseri + C. difficile* mice (Fig. 5B; Fig. S4B).

Until relatively recently, the *Muribaculaceae* (previously known as S24-7 [83]) have been understudied due to the absence of a suitable culture medium that supported their growth (84), resulting in a paucity of functional studies investigating their biology (85). Nonetheless, bioinformatic and genomic analysis of these bacteria highlights their ubiquitous nature in the mammalian intestinal microbiota and their expanded capacity for glycan catabolism (84). Notably, a recent study has drawn a link between the *Muribaculaceae* and their ability to sequester the host glycans that *C. difficile* prefers, leading to their use within a defined bacterial consortium capable of providing colonization resistance against *C. difficile* via nutrient competition (86).

Our data suggest that several members of this family have the potential to protect against *C. difficile* colonization, so we hypothesized that they may be able to directly inhibit or compete with *C. difficile* for nutrients. To test this, we co-cultured two members of the *Muribaculaceae* from different genera, *Duncianiella muris* DSM 103720 and *Muribaculum intestinale* DSM 28989, based on the predicted genera of ASV17, ASV43, and ASV48. These species were grown in the rich medium fastidious anaerobe broth (FAB) that we supplemented with additional vitamins and nutrients. Despite the rich nutrient conditions, these bacteria grow considerably slower than *C. difficile* in monoculture. To model the dynamics of the incoming pathogen *C. difficile* with the existing *Muribaculaceae* and replicate what a previous group did when co-culturing these bacteria, we staggered the co-cultures by first inoculating them with either *D. muris* or *M. intestinale*. Then after 12 h of growth, we inoculated them with *C. difficile* (86). Given their reported role in sugar competition with *C. difficile* (86), we supplemented our media with either 1% N-acetylneuraminic acid (Neu5Ac) or 1% N-acetylglucosamine (GlcNAc), two prominent host-derived monosaccharides.

**Fig 6:**
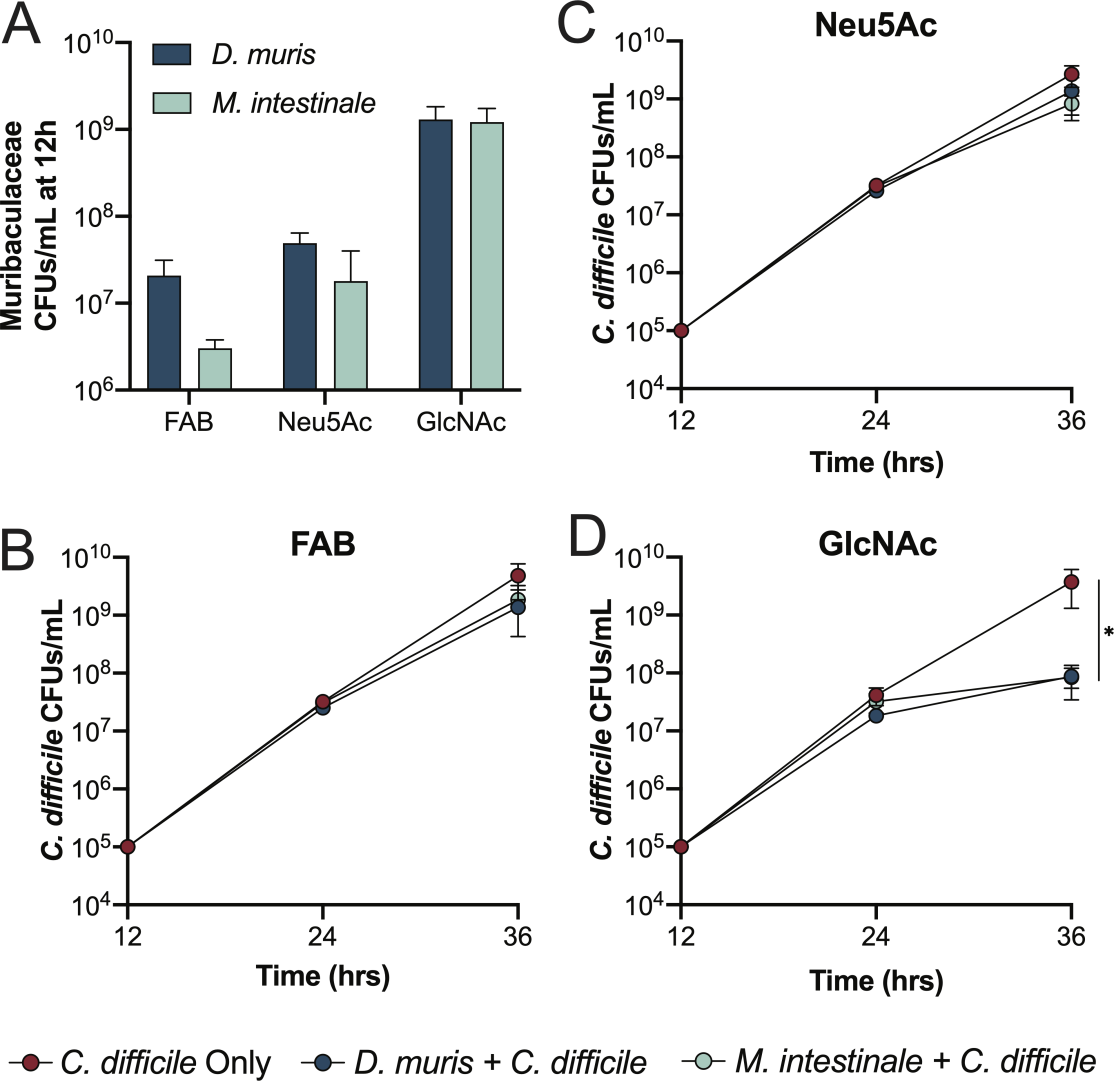
*Muribaculaceae* can restrict *C. difficile in vitro*. (**A**) *D. muris* and *M. intestinale* growth after 12 h in FAB with or without 1% Neu5Ac or GlcNAc. (**B–D**) Growth of *C. difficile* in co-cultures. Individual *Muribaculaceae* species grew alone for 12 h before *C. difficile* was inoculated and grown until 36 h. Co-cultures took place in (**B**) FAB, (**C**) FAB + 1% Neu5Ac, and (**D**) FAB + 1% GlcNAc. All data presented as mean ± standard deviation from *n* = 4 replicates. Kruskal-Wallis test was used to determine statistical significance. * *P* < 0.05.

Both *D. muris* and *M. intestinale* grew robustly in mono-culture in FAB supplemented with GlcNAc after 12 h compared to the FAB alone and Neu5Ac conditions (Fig. 6A). All cultures were subsequently inoculated with 10^5^ CFUs/mL of *C. difficile* to measure competition (Fig. 6B through D). After 24 h, we were not able to recover any viable *Muribaculaceae* from co-cultures, suggesting that *C. difficile* has some mechanism to antagonize these commensal species. Nonetheless, *C. difficile* growth was significantly inhibited in both co-cultures in which the *Muribaculaceae* grew in the presence of GlcNac (Fig. 6D). While it remains unclear whether this inhibition was due to the production of antagonistic metabolites or if it competed with *C. difficile* for shared nutrients, these data suggest that robust *Muribaculaceae* growth can result in the restriction of *C. difficile in vitro*. Given our observation that a *Muribaculaceae* bloom in *L. gasseri + C. difficile* mice coincided with the return of colonization resistance at week 3 (Fig. 5A and B), these *in vitro* co-cultures may be modeling some of the dynamic interactions that led to the *C. difficile* inhibition *in vivo*. Furthermore, these data highlight the need to reevaluate existing probiotic species in the context of disease as well as identify and investigate new species as candidates for the next generation of probiotics, with both direct and indirect effects needing to be determined.

## DISCUSSION

Over the last 40 years, CDI has been the leading cause of AAD and colitis (87).

Probiotics have been repeatedly touted as a solution for AAD and CDI (88–90). Yet, we know relatively little about how probiotics interact with the gut microbiota and host after antibiotic treatment. While several human trials support probiotic administration as a strategy to prevent or treat CDI (91–95), others suggest that they do not improve CDI outcomes (36, 96). Translational models of CDI have been subject to these inconsistencies as well. For example, *L. acidophilus* GP1B was shown to decrease *C. difficile* in a mouse model of CDI (62), and *L. acidophilus* La-5 demonstrated *C. difficile* inhibition *in vitro* (97). Based on their low efficacy and the many discrepancies surrounding probiotic research, the American College of Gastroenterology guidelines do not support the use of probiotics in CDI patients receiving antibiotic therapy (98). The unpredictability and inadequacies of probiotics in the clinic are the result of several factors and compounded by the fact that probiotic trial design is remarkably difficult, given that differences in dose/timing of administration, the species/strain selected, and a single strain or a multi-strain formulation are all variables that impact clinical outcomes.

Here, we investigated the impact of a single dose of *L. acidophilus* NCFM or *L. gasseri* Lg-36 after cefoperazone treatment in a mouse model of CDI to determine whether these probiotic strains can alter colonization resistance against *C. difficile*. The single dose allowed us to accurately track the colonization of both species and demonstrated that their colonization of the mouse gut was transient (Fig. 2A), likely due to them becoming excluded by members of the indigenous microbiota (99). Nonetheless, our experiment can be categorized into the weeks 1 and 2, where probiotic strains were present and able to directly interact with the microbiota short-term, and weeks 3 and 4, where they were no longer detectable and yet their administration imparted a long-term indirect effect on the microbial community. This lasting impact from temporary probiotics can result in a “microbiome scar” with potentially significant consequences for human health (41).

We found that *L. acidophilus + C. difficile* mice had an increased burden of *C. difficile* at weeks 2 and 3 following antibiotic exposure (Fig. 2B). *L. acidophilus* was present in the mouse cecum at week 2 and, therefore, could directly support *C. difficile* infection. While we did not explore the microbial interactions between *L. acidophilus* and *C. difficile* in this study, *Lactobacillaceae* have previously been associated with CDI in patients and in mice (100, 101), suggesting that members of this family may enhance *C. difficile* fitness similar to the related lactic acid bacteria *Enterococcus* (102). *L. acidophilus* has high proteolytic activity (103), which may support the cross-feeding of critical amino acids, such as ornithine (15), to *C. difficile* and provide a potential mechanism that explains the increased infection (102, 104). Compared to the potential for direct interactions between *L. acidophilus* and *C. difficile*, *L. acidophilus*’ absence by week 3 suggests that its impact on colonization resistance may have occurred indirectly by impairing the reconstitution of the indigenous microbiota. The production of soluble factors, such as bacteriocins by *L. acidophilus*, known to produce the class III bacteriocin lactacin B (105), and other probiotic strains can impair the reconstitution of the human fecal microbiota following antibiotic treatment and shape the composition of bacterial communities (41, 106). Here, *L. acidophilus* may have altered microbial succession and increased susceptibility to CDI similarly through the secretion of soluble factors that do not inhibit *C. difficile* (Fig. 3A). Given the prominence of *L. acidophilus* in probiotic formulations, these findings are noteworthy and highlight the potential unintended consequences of probiotics. Furthermore, these results provide insight into the potential negative effects of specific probiotic strains in hospital settings or after antibiotic treatments.

In contrast to *L. acidophilus*, *L. gasseri + C. difficile* mice displayed a lower burden of *C. difficile* at weeks 1, 2, and 3 (Fig. 2B). In weeks 1 and 2, *L. gasseri* was present in sufficient levels to directly interact with *C. difficile* and inhibit it. *L. gasseri* is able to produce a soluble factor that was consistent with a bacteriocin to inhibit *C. difficile in vitro* (Fig. 3A) (68). While this putative bacteriocin completely inhibited *C. difficile in vitro*, high levels of *L. gasseri* colonization were not sufficient to prevent CDI disease in week 1 of the experiment (Fig. 2A and B), suggesting that antimicrobial production and inhibition *in vivo* may not be as straightforward to achieve as it is *in vitro*. Probiotic strains of *L. gasseri* are commonly thought to improve CDI outcomes in part due to the direct inhibition of *C. difficile* through several mechanisms (107). Short-chain fatty acids (SCFAs) and bile acids (BAs) are the product of microbial metabolism and known to shape the gut microbiota and inhibit *C. difficile* (108, 109). Probiotics can shape SCFA and BA profiles with the potential to impact CDI (110–113). Antimicrobial bacteriocins, such as reuterin, produced by *L. reuteri* have been proposed as an adjunct therapy for CDI because of its inhibition of *C. difficile* (54). Similarly, production of the bacteriocin Gassericin and by *L. gasseri* has the potential to inhibit *C. difficile* (74). Broad-spectrum antimicrobials and metabolites that modulate the microbiota may be a viable strategy to inhibit enteric pathogens, but their lack of specificity could also result in further perturbation to the gut microbiome, worsening AAD or initiating CDI recurrence (114, 115).

The other probiotic function thought to improve CDI outcomes is via the reconstitution of the gut microbiota (107). Yet, our understanding of this function is incomplete, thereby limiting the translation of probiotic research into the clinic. Rationally and precisely manipulating the microbiota given its complexity and variability is a feat that has only been accomplished through FMT (116), though deliberately reconstituting selective functions of the microbiome, such as BA metabolism, has been achieved to target *C. difficile* (117). Given these challenges and the complexities of FMTs, new probiotic strains labeled as “next-generation” probiotics are sought after as a way to overcome the functional limitations of existing single strains (54, 118), though their discovery and identification in the context of CDI can be laborious (119).

While relatively few studies have focused on the *Muribaculaceae* family until recently, these bacteria colonize mammals, including humans, and are typically associated with health, making strains in this family potential next-generation probiotic candidates (84). This family, including the *Duncaniella* and *Muribaculum* genera, harbors many carbohydrate-active enzymes that support their catabolism of host and dietary glycans and the production of SCFAs (84, 120). Because of their capacity to forage host glycans, *Muribaculaceae* have been leveraged to treat CDI by competing with *C. difficile* for sugars like GlcNAc (86, 121), a monosaccharide found on secreted mucins that can promote *C. difficile* colonization (122, 123). Our finding that GlcNAc boosts *M. intestinale* and *D. muris* growth *in vitro*, resulting in restricted *C. difficile* growth, supports the hypothesis that the *Muribaculaceae* promote colonization resistance *in vivo* (Fig. 5D), though the mechanisms that mediate this effect are not clear. Nonetheless, these results support the need for additional studies to functionally investigate the role of *Muribaculaceae* in gut health.

We observed members of the *Muribaculaceae* enriched in *L. gasseri + C. difficile* mice with colonization resistance against *C. difficile* at week 3 (Fig. 4B). This *Muribaculaceae* bloom occurred after *L. gasseri* was no longer detectable, suggesting that *L. gasseri* created a niche for *Muribaculaceae* to expand into. However, it is also reasonable to assume that this bloom occurred serendipitously in these mice. *Muribaculaceae* species are not always found inhabiting genetically identical mice, and their stochastic presence has been implicated in the variability of mouse models of disease (81). Starting microbiome features, such as the presence of key species, have been shown to dictate colonization resistance in experiments (99), and this stochasticity in microbiome studies presents a challenge for researchers to reliably analyze and reproduce data (124). Additionally, our results arise from the very specific timing and dosing of gavages outlined in our methods, thereby making replicability a difficult task. Although we are limited by the number of mice that demonstrate our observations from week 3 *L*. *gasseri + C. difficile* condition, we believe the results of this single timepoint can be viewed as an ecological case study that was modeled and validated *in vitro* to make our interpretations robust (Fig. 6). Considering our overall experiment contained multiple timepoints when *L. gasseri* inhibited *C. difficile* post-antibiotics, our integrated findings are likely generalizable. In a broader context, this can provide a framework for teasing apart different ecological mechanisms that drive different probiotic-associated phenotypes.

Antibiotic-induced perturbations to the gut microbiota can result in the loss of colonization resistance against pathogens like *C. difficile*, and probiotic strains similarly have the potential to colonize and exacerbate the antibiotic-associated dysfunction of the microbiota by impairing its reconstitution. Despite the potential for risk by adversely impacting the indigenous microbiota, *Lactobacillus* probiotics are a valuable and diverse set of tools to interrogate and address gut health. In this study, *L. acidophilus* enhanced CDI, while *L. gasseri* resisted it, though its probiotic activity was still not effective enough to dampen disease (Fig. 2C; Fig. S1). This observation supports the potential use of specific *L. gasseri* strains as adjunct treatment to support the reconstitution of the microbiome and discourage CDI recurrence and *C. difficile* carriage, which can contribute to the spread of the disease in hospital environments (125). Given the opposing effects of these two related strains, our work emphasizes how individual probiotics need to be well examined or even engineered in order to ensure safety and determine efficacy (126) both in terms of their direct impact on the microbiome and their indirect lasting effect after colonization has dwindled. Additionally, the role of the *Muribaculaceae* in the gut has remained elusive, but studies investigating the members of this family are critical to discover and understand their function. Given our findings, we stress the need for more long-term assessments of probiotic effects on the gut microbiota and for the evaluation of strains indigenous to the gut microbiota as next-generation probiotic candidates in order to improve the current preventative and therapeutic strategies to address AAD and CDI.

## MATERIALS AND METHODS

### Bacterial strains and growth conditions

*Lactobacillus* species were cultured statically in MRS broth. Spontaneous rifampicin-resistant clones of *Lactobacillus* strains were created as previously described and enumerated on selective LBS agar containing 100 µg/mL rifampicin (65). *C. difficile* R20291 was statically cultured anaerobically using BHIS broth supplemented with 100 mg/L of L-cysteine. *C. difficile* R20291 enumeration was performed on TCCFA (cefoxitin, cycloserine, and fructose agar with 0.1% TCA). *Muribaculaceae* were cultured in fastidious anaerobe broth (Neogen) or agar supplemented with 5.8 µM vitamin K_3_, 1.44 µM 139 FeSO_4_⋅7H_2_O, 1 mM MgCl_2_, 1.9 µM hematin, 0.2 mM L-histidine, 3.69 nM vitamin B_12_, 208 140 µM L-cysteine, and 7.2 µM CaCl_2_⋅2H_2_O. All agar plates were made using 1.5% agar. *Muribaculaceae-C. difficile* co-cultures were supplemented with 1% w/v sugars, and growths were carried out in clear 96-well flat-bottom plates containing 200 µL of media per well. Growths were performed in a Tecan plate reader within the anaerobic chamber at 37°C for 36 h.

### Spore preparation

*C. difficile* spores were prepared as previously described (127, 128). Briefly, *C. difficile* was grown at 37°C anaerobically for 1 week in Clospore media (129). Spores were harvested by centrifugation and washed with water, heat-treated for 20 min at 65°C, and stored at 4°C. Spores were plated on BHIS and TBHIS to make sure no viable cells were present.

### Animals and housing

Animal experiments were conducted in the Laboratory Animal Facilities located on the North Carolina State University (NCSU) College of Veterinary Medicine (CVM) campus. The animal facilities are equipped with a full-time animal care staff coordinated by the Laboratory Animal Resources division at NCSU. The NCSU CVM is accredited by the Association for the Assessment and Accreditation of Laboratory Animal Care International. Trained animal handlers in the facility fed and assessed the status of animals several times per day.

### Mouse model of *C. difficile* infection

Six- to eight-week-old male mice housed four mice/cage were given 0.5 mg/mL cefoperazone in their drinking water for 5 days to make them susceptible to *C. difficile* infection (64). Subsequently, mice were then given normal water (Gibco) for 2 days. Mice were orally gavaged with 10^8^ CFUs of *L. acidophilus* or *L. gasseri*, and each cage of mice was then challenged at day 0 with 10^5^ *C. difficile* spores and at each week for 4 weeks total. Mice were weighed daily and monitored for clinical signs of distress (ruffled fur, hunched posture, slow ambulation). After 4 days post-*C. difficile* challenge, mice were humanely sacrificed, and necropsy was performed. Fecal and cecal contents were freshly harvested for enumeration of *C. difficile* CFUs using TCCFA and *Lactobacillus* CFUs using LBS agar with 100 µg/mL rifampicin. Cecal and fecal contents collected for sequencing or cytotoxicity assays were flash-frozen in liquid N_2_.

### Vero cell cytotoxicity assay

Vero cells were grown and maintained in Dulbecco’s modified Eagle medium (DMEM) supplemented with 10% fetal bovine serum and 1% penicillin streptomycin solution. Harvested cells were collected by incubating with 0.25% trypsin, washing with 1× DMEM, and centrifuging at 1,000 rpm for 5 min. Cells were plated at 1 × 10^4^ cells per well in a 96-well flat bottom microtiter plate and incubated overnight at 37°C/5% CO_2_. Cecal contents were serially diluted and incubated 1:1 with PBS or antitoxin for 40 min at 25°C. Diluted samples were added to the Vero cells and incubated overnight at 37°C/5% CO_2_. After incubation, cell rounding was visualized under 200× magnification on a microscope. The cytotoxic titer was defined as the reciprocal of the highest dilution that produced rounding in 80% of Vero cells for each sample. Vero cells treated with purified *C. difficile* toxins (A and B) and antitoxin were used as controls.

### Histopathological examination of the mouse cecum

At the time of necropsy, tissue from the cecum and colon was prepared for histology by placing the intact tissue into histology cassettes and storing in 10% buffered formalin for 48 h at room temperature, then transferring to 70% ethyl alcohol for long-term storage. Tissues were processed, paraffin-embedded, sectioned at 4 µm thickness, and hematoxylin and eosin-stained for histopathological examination (University of North Carolina Animal Histopathology & Lab Medicine core). Histological specimens were randomized and scored in a blinded manner by a board-certified veterinary pathologist. Edema, inflammation (cellular infiltration), and epithelial damage for the cecum and colon were each scored 0–4 based on a previously published numerical scoring scheme (64). Edema scores were as follows: 0, no edema; 1, mild edema with minimal (2×) multifocal submucosal expansion or a single focus of moderate (2–3×) submucosal expansion; 2, moderate edema with moderate (2–3×) multifocal submucosal expansion; 3, severe edema with severe (3×) multifocal submucosal expansion; 4, same as score 3 with diffuse submucosal expansion. Cellular infiltration scores were as follows: 0, no inflammation; 1, minimal multifocal neutrophilic inflammation of scattered cells that do not form clusters; 2, moderate multifocal neutrophilic inflammation (greater submucosal involvement); 3, severe multifocal to coalescing neutrophilic inflammation (greater submucosal ± mural involvement); 4, same as score 3 with abscesses or extensive mural involvement. Epithelial damage was scored as follows: 0, no epithelial changes; 1, minimal multifocal superficial epithelial damage (vacuolation, apoptotic figures, villus tip attenuation/necrosis); 2, moderate multifocal superficial epithelial damage (vacuolation, apoptotic figures, villus tip attenuation/necrosis); 3, severe multifocal epithelial damage (same as above) +/− pseudomembrane (intraluminal neutrophils, sloughed epithelium in a fibrinous matrix); 4, same as score 3 with significant pseudomembrane or epithelial ulceration (focal complete loss of epithelium).

### 16S rRNA Illumina sequencing and microbiome analysis

DNA was isolated from feces and cecal snips at the University of Michigan Microbial Systems Molecular Biology Laboratory. The Mag Attract Power Microbiome Kit (Mo Bio Laboratories, Inc.) was used to isolate DNA from cecal snips and feces. Dual-indexing sequencing approach was used to amplify the V4 region of the 16 S rRNA gene. Each PCR mixture contained 2 µL of 10× Accuprime PCR buffer II (Life Technologies, CA, USA), 0.15 µL of Accuprime high-fidelity polymerase (Life Technologies, CA, USA), 5 µl of a 4.0 µM primer set, 1 µL DNA, and 11.85 µL sterile nuclease-free water. The template DNA concentration was 1 to 10 ng/µL for a high bacterial DNA/host DNA ratio. The PCR conditions were as follows: 2 min at 95°C, followed by 30 cycles of 95°C for 20 s, 55 °C for 15 s, and 72°C for 5 min, followed by 72°C for 10 min. Libraries were normalized using a Life Technologies SequalPrep Normalization Plate Kit as per the manufacturer’s instructions for sequential elution. The concentration of the pooled samples was determined using the Kapa Biosystems Library Quantification Kit for Illumina platforms (Kapa Biosystems, MA, USA). Agilent Bioanalyzer High-Sensitivity DNA Analysis Kit (Agilent CA, USA) was used to determine the sizes of the amplicons in the library. The final library consisted of equal molar amounts from each of the plates normalized to the pooled plate at the lowest concentration. Sequencing was done on the Illumina MiSeq platform using a MiSeq Reagent Kit V2 (Illumina, CA, USA) with 500 cycles according to the manufacturer’s instructions, with modifications (130). Sequencing libraries were prepared according to Illumina’s protocol for preparing libraries for sequencing on the MiSeq (Illumina, CA, USA) for 2 or 4 nM libraries. PhiX and genomes were added in 16S amplicon sequencing to add diversity. Sequencing reagents were prepared according to the Schloss SOP (https://www.mothur.org/wiki/MiSeq_SOP#Getting_started), and custom read 1, read 2, and index primers were added to the reagent cartridge. FASTQ files were generated for paired-end reads. Raw reads were processed in R v4.4.1, with DADA2 v1.32.0 used for de-noising and generating amplicon sequence variants (ASVs) (131). Taxonomic assignment of the ASVs was done using the Silva reference database version 138.1 (132). The code used to process and analyze the reads can be found at DOI: 10.5281/zenodo.13850729. All ASV sequences and taxonomic information are listed in Table S1. Inverse Simpson diversity and NMDS with a maximum iteration of 5 and 50 random starts were determined using PhyloSeq R package v1.48.0. Pairwise Adonis for beta diversity was performed using the pairwiseAdonis R package v0.4.1 using 999 permutations, and FDR correction Kruskal-Wallis and Dunn tests with FDR correction were performed using rstatix R package v0.7.2. Differential abundance analysis on week 3 cecum samples was performed using ALDEx2 R package v1.36.0. Briefly, the ALDEX2 package was used to generate generalized linear models using the center log ratio-transformed Monte Carlo Dirichlet instances of the counts of each ASV to determine changes relative to *L. gasseri + C. difficile*.

### Statistics

All statistical analyses were performed in GraphPad Prism 9. All data met the assumptions of the statistical tests used, and data distribution was assumed to be normal in log_10_-transformed values, but this was never formally tested. Data collection was not randomized, and analysis was not blinded. No data were excluded from analysis. All graphed bars represent mean  ±  s.d. Asterisks indicate significant differences (*P  <  0.05, **P  <  0.01, ***P  <  0.001, ****P <  0.0001).

## Data Availability

All data associated with this study are available in the main text or the supplemental material. Raw 16S sequencing files are available in NCBI under BioProject no. PRJNA1166207.

## SUPPLEMENTAL MATERIAL

Figure S1 mbio.01468-25-s0001.pdf Mouse weight change after *C. difficile* challenge by week.

Figure S2 mbio.01468-25-s0002.pdf Histopathological changes to the murine cecum after *C. difficile* challenge.

Figure S3 mbio.01468-25-s0003.pdf *Lactobacillus* administration alters fecal microbial diversity after *C. difficile* challenge.

Figure S4 mbio.01468-25-s0004.pdf *Lactobacillus* administration results in unique taxonomic differences in the feces after antibiotic treatment.

Supplemental legends mbio.01468-25-s0005.docx Legends for the supplemental figures and Table S1.

Table S1 mbio.01468-25-s0006.xlsx Taxonomic and sequence information.

ASM does not own the copyrights to Supplemental Material that may be linked to, or accessed through, an article. The authors have granted ASM a non-exclusive, world-wide license to publish the Supplemental Material files. Please contact the corresponding author directly for reuse.
