# Supplementary figures and images for: Differential modulation of post-antibiotic colonization resistance to Clostridioides difficile by two probiotic Lactobacillus strains
Source: mBio. 2025 Jul 21;16(8):e01468-25. doi: 10.1128/mbio.01468-25 (PMC12345269; doi:10.1128/mbio.01468-25)

**Week 1**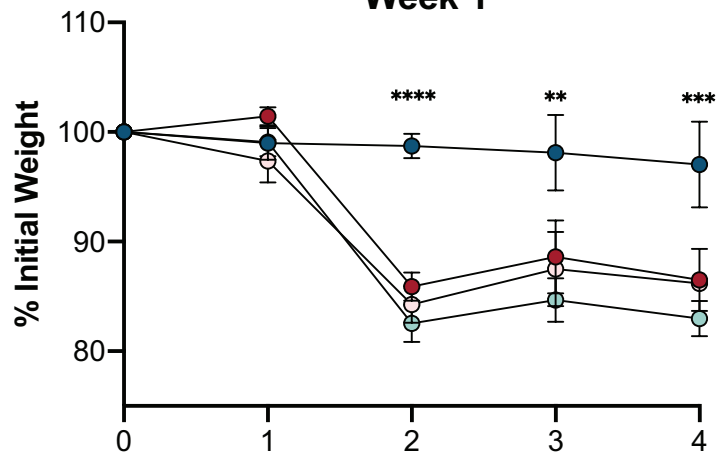**Week 3**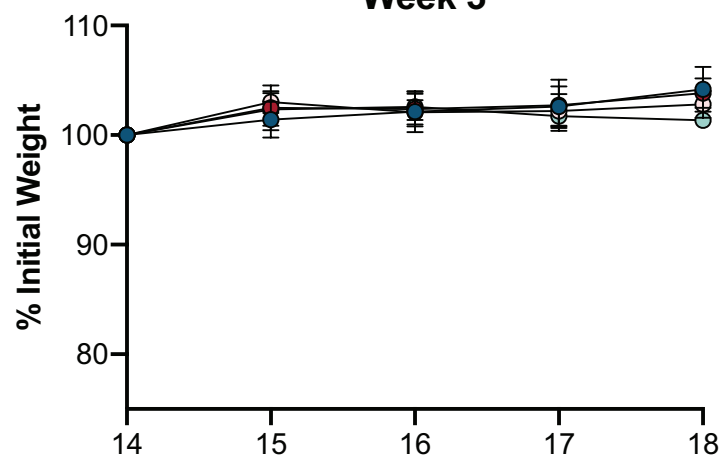**Week 2**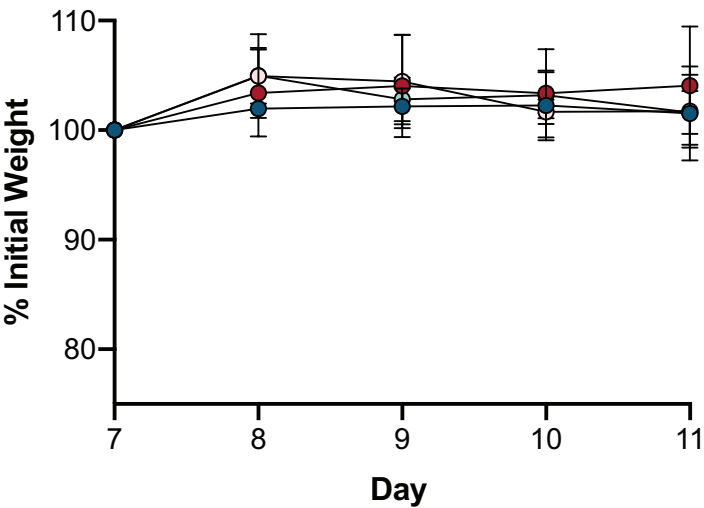**Week 4**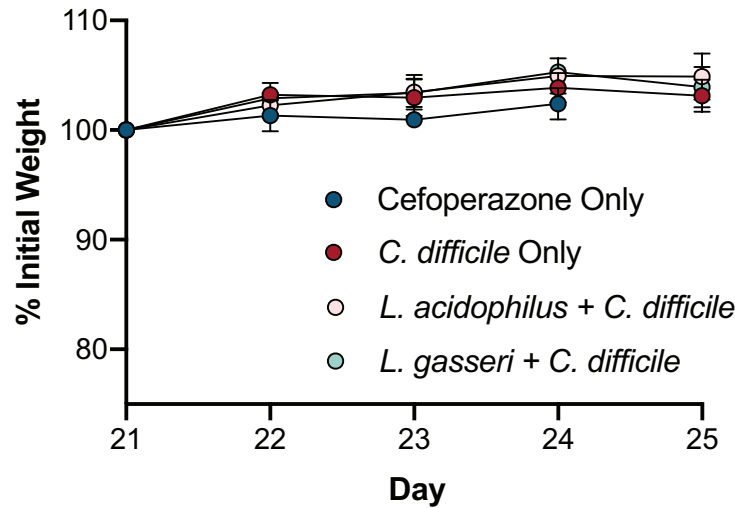

Supplement: Figure S1 — Mouse weight change after C. difficile challenge by week. [file mbio.01468-25-s0001.pdf]

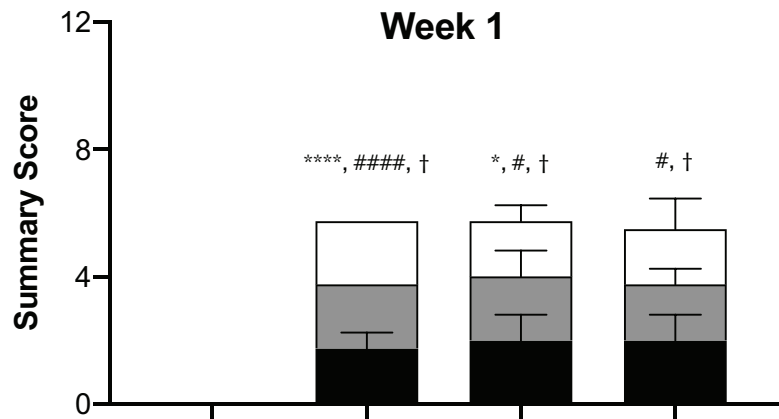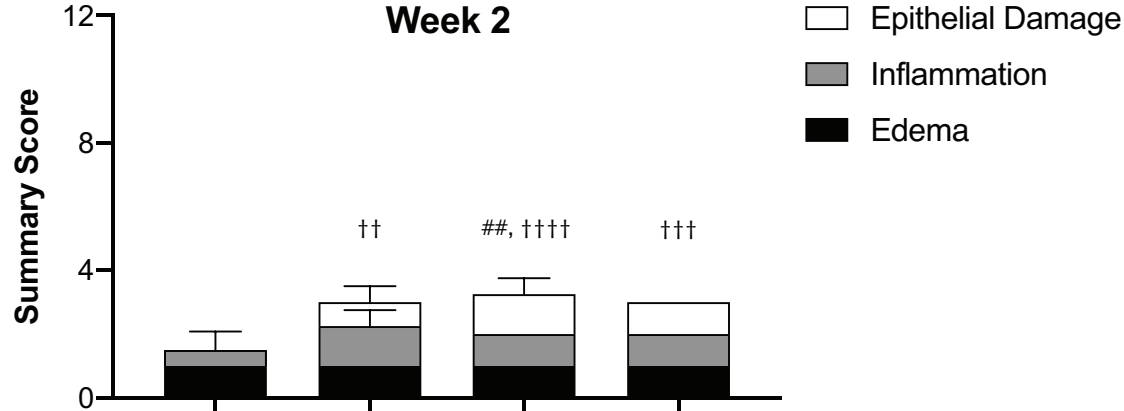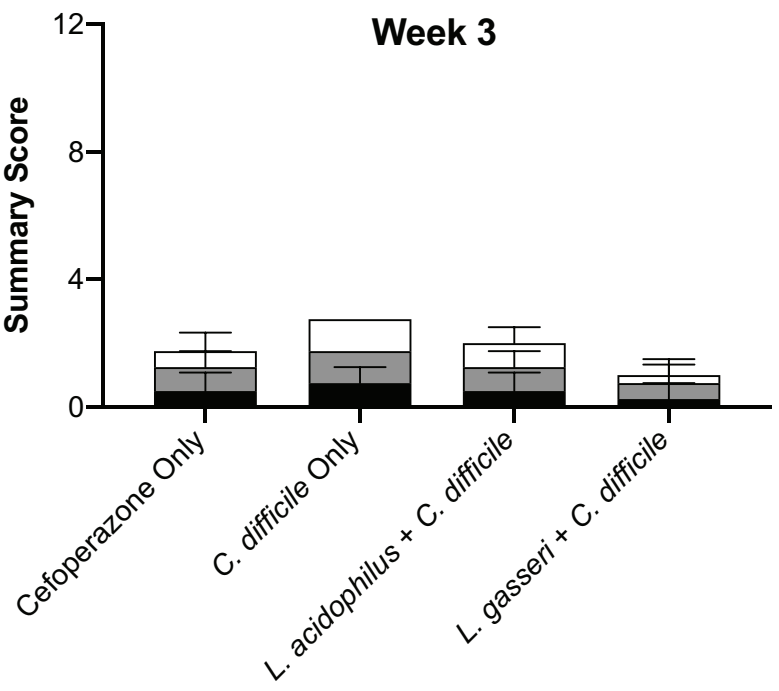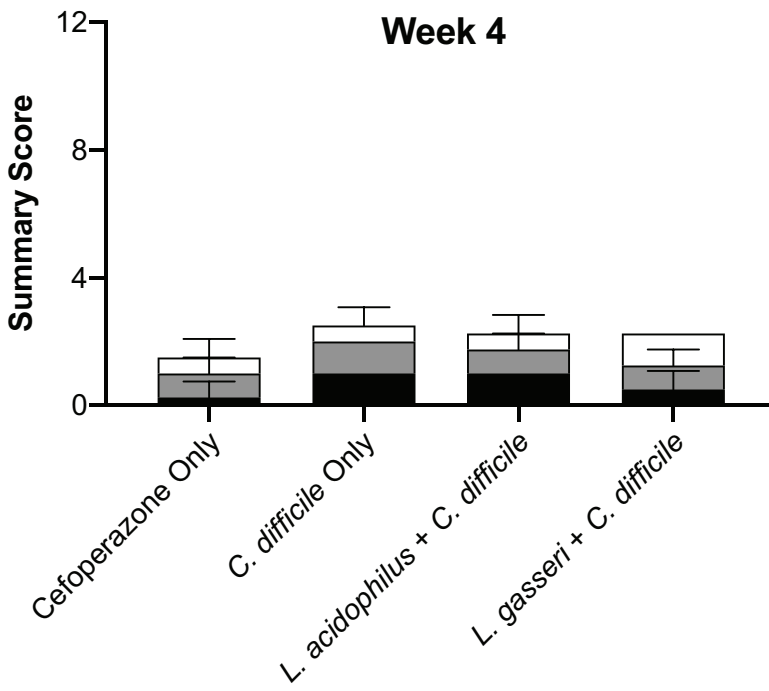

Supplement: Figure S2 — Histopathological changes to the murine cecum after C. difficile challenge. [file mbio.01468-25-s0002.pdf]

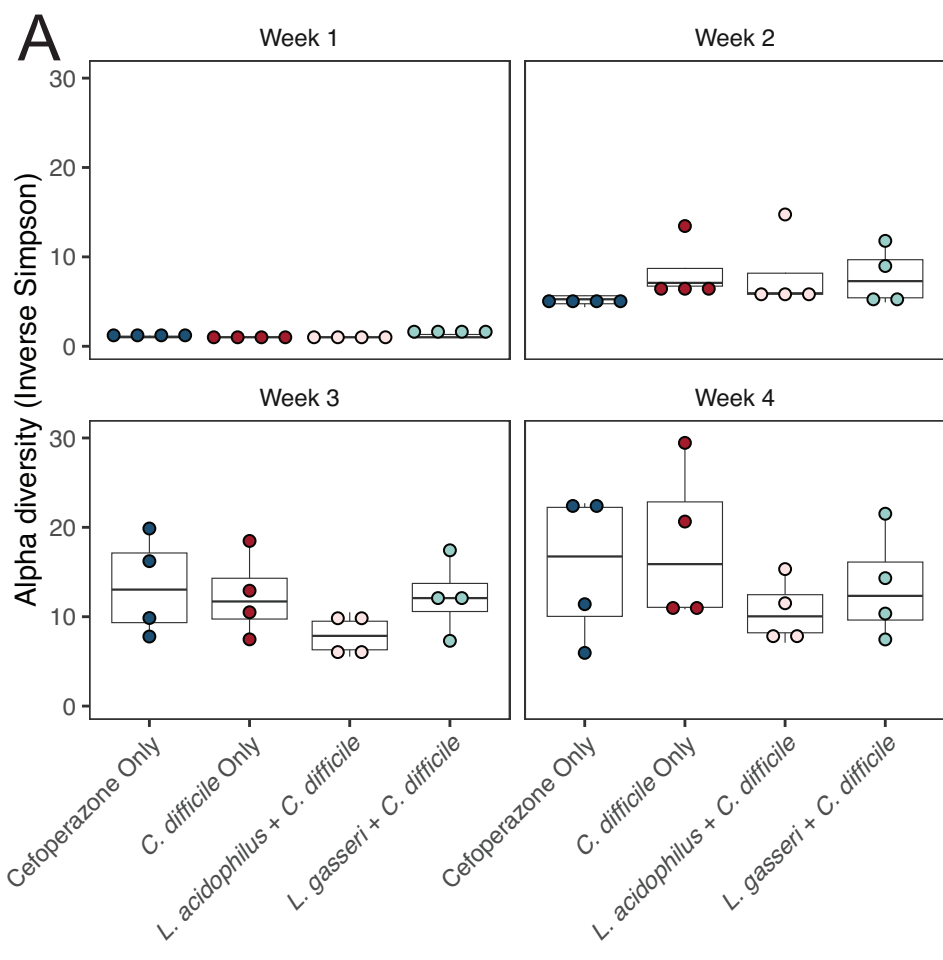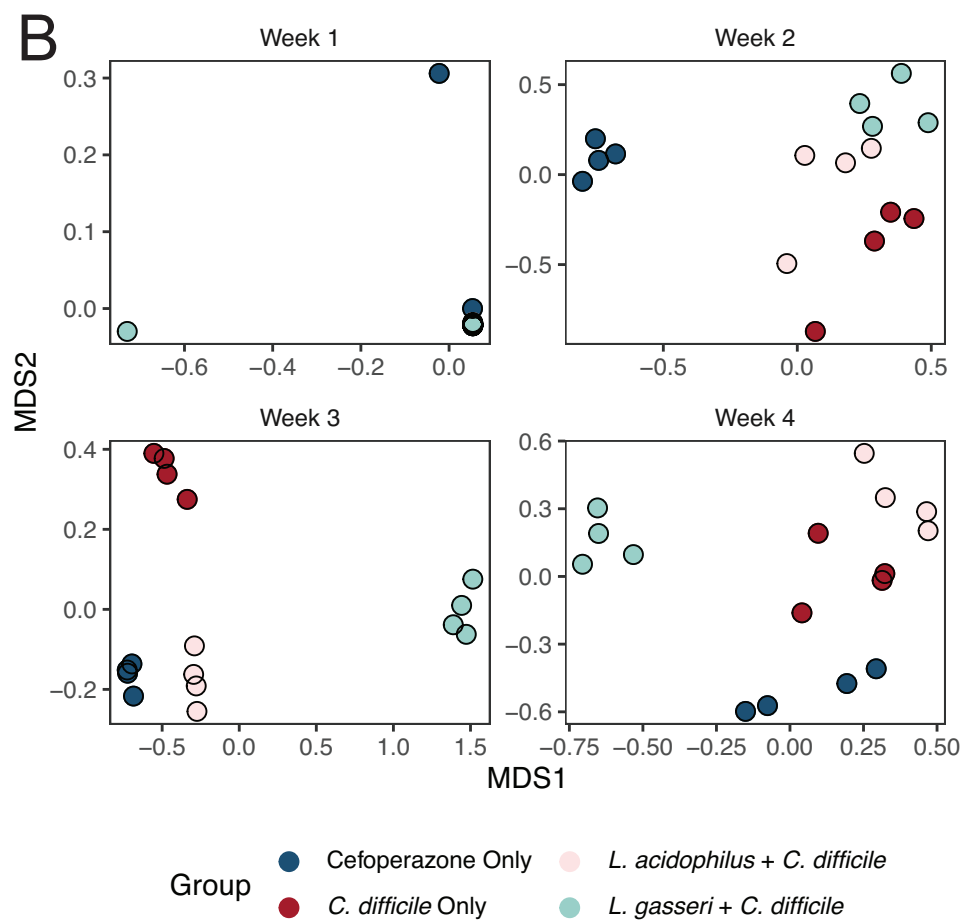

Supplement: Figure S3 — Lactobacillus administration alters fecal microbial diversity after C. difficile challenge. [file mbio.01468-25-s0003.pdf]

A

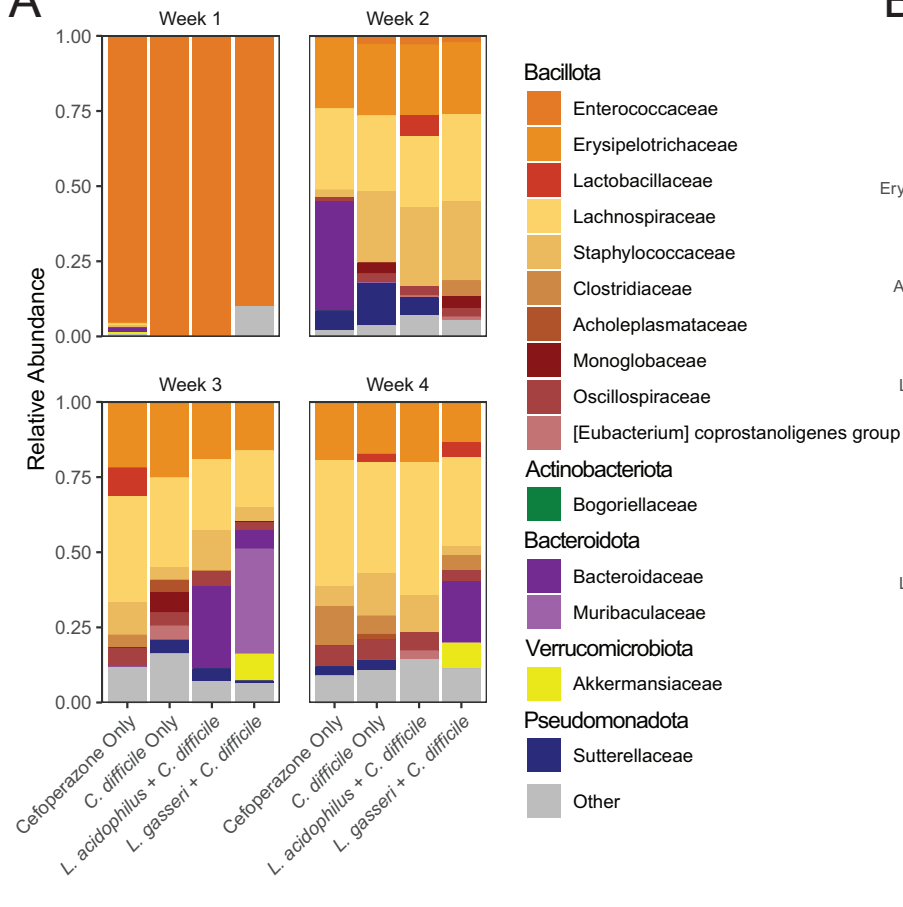

B

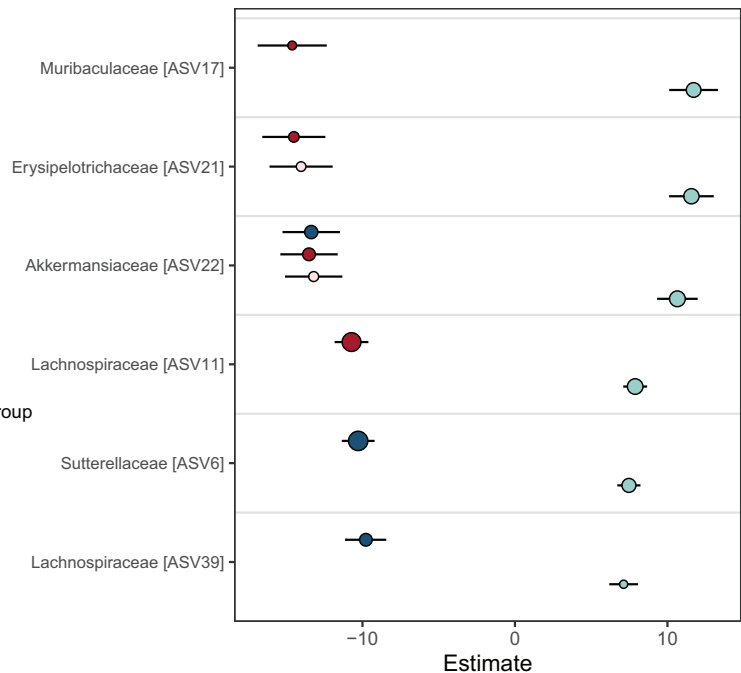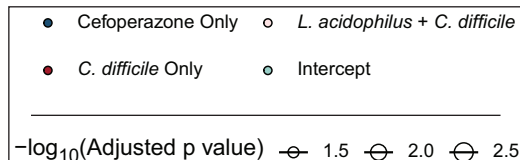

Supplement: Figure S4 — Lactobacillus administration results in unique taxonomic differences in the feces after antibiotic treatment. [file mbio.01468-25-s0004.pdf]
