# Supplementary material for: Differential modulation of post-antibiotic colonization resistance to Clostridioides difficile by two probiotic Lactobacillus strains
Source: mBio. 2025 Jul 21;16(8):e01468-25. doi: 10.1128/mbio.01468-25 (PMC12345269; doi:10.1128/mbio.01468-25)
Supplement: Supplemental legends — Legends for the supplemental figures and Table S1. [file mbio.01468-25-s0005.docx]

**Supplemental legends**

**Figure S1. Mouse weight change after *C. difficile* challenge by week**. Percent baseline weights for mice in each group displayed by each week. All data points presented as mean ± standard deviation of n= 4 mice. A One-Way ANOVA with Tukey’s correction for multiple comparisons was used to determine statistically significant weight change. ***p* < 0.01, ****p* < 0.001, *****p* < 0.0001.

**Figure S2. Histopathological changes to the murine cecum after *C. difficile* challenge**. Histopathological summary scores of the cecum. All stacked bars are presented as mean ± standard deviation. The symbols *, #, and † indicate statistical significance for comparisons made between epithelial damage, inflammation, and edema, respectively. A Two-Way ANOVA with Tukey’s correction for multiple comparisons was used to determine significance. All significant differences were found to be between Cefoperazone only mice and the other groups. **p* < 0.05, ***p* < 0.01, ****p* < 0.001, *****p* < 0.0001, with the number of asterisks used applicable to the number of the other symbols to indicate significance too.

**Figure S3. *Lactobacillus* administration alters fecal microbial diversity after *C. difficile* challenge** (A) Inverse Simpson index of ASVs in fecal microbiotas presented in box and whisker plots for each week. No significant differences as determined by Kruskal-Wallis were observed. (B) Bray-Curtis dissimilarity of cecal ASVs between samples plotted by NMDS. The stress of each NMDS is listed in the top corner of each graph.

**Figure S4.** ***Lactobacillus* administration results in unique taxonomic differences in the feces after antibiotic treatment.** (A) Relative abundance of ASVs from the fecal microbiota grouped by bacterial families each week post antibiotics. (B) Estimates obtained from generalized linear model with holm correction of center log ratio transformed Monte Carlo Dirichlet instances obtained from ALDEx differential-abundance analysis relative to ASVs in the *L. gasseri* + *C. difficile* group. The intercept serves as a reference for the *L. gasseri* + *C. difficile* group to show differences between this group and estimates from other groups. Only significant (*p* adjusted ≤ 0.05) points are plotted.

**Supplemental Table 1. ASV Taxonomic and sequence information.**
